# Farm typology for planning targeted farming systems interventions for smallholders in Indo-Gangetic Plains of India

**DOI:** 10.1038/s41598-021-00372-w

**Published:** 2021-10-25

**Authors:** Jashanjot Kaur, A. K. Prusty, N. Ravisankar, A. S. Panwar, M. Shamim, S. S. Walia, S. Chatterjee, M. L. Pasha, Subhash Babu, M. L. Jat, Santiago López-Ridaura, Jeroen C. J. Groot, Roos Adelhart Toorop, Luis Barba-Escoto, Kohima Noopur, Poonam Kashyap

**Affiliations:** 1 School of Organic Farming, Punjab Agricultural University, Ludhiana, India; 2 ICAR-Indian Institute of Farming Systems Research (ICAR-IIFSR), Modipuram, India; 3 Department of Agricultural Economics, Bidhan Chandra Krishi Vishwavidyalaya, Kalyani, Nadia, WB 741 235 India; 4 College of Agriculture, PJTSAU, Rajendranagar, Hyderabad, Telangana 500 030 India; 5 Division of Agronomy, ICAR-Indian Agricultural Research Institute, New Delhi, 110012 India; 6 International Maize and Wheat Improvement Center (CIMMYT), New Delhi, India; 7 International Maize and Wheat Improvement Center (CIMMYT), El-Batan, Mexico; 8 Farming Systems Ecology, Wageningen University and Research, Wageningen, The Netherlands; 9 Sher-e-Kashmir University of Agricultural Sciences and Technology of Jammu, Jammu, India

**Keywords:** Systems analysis, Climate-change adaptation, Socioeconomic scenarios, Sustainability

## Abstract

Due to complexity of smallholder farms, many times technologies with great potential fail to achieve the desired impact in leveraging productivity and profitability of the farming community. In the Indo-Gangetic Plains there is an urgent need to understand the diversity of farm households, identifying the main drivers deciding their system thus, classifying them into homogenous groups. In the present study, the diversity of smallholder farms was assessed using crop, livestock and income related characteristics and associated farm mechanization. Using principal component analysis and cluster analysis for 252 farm households, 4 farm types were identified i.e. Type 1. Small Farm households with cereal-based cropping system and subsistence livestock (39%), Type 2. Small Farm households with diversified cropping system dominated by cereal and fodder crops with only cattle herd (9%), Type 3. Marginal Farm household with diversified cropping system dominated by cash crop and herd comprising of only cattle (39%), Type 4. Marginal Farm household with diversified cropping system dominated by cereal crops and herd dominated by small ruminants (12%). Based on the constraints identified for different components of farming systems, low-cost interventions were planned for each farm type. These interventions have resulted in 84.8–103.2 per cent increase in the income of the farm HH under study suggesting usefulness of typology-based intervention planning in increasing income of small farm holders.

## Introduction

Agriculture is the core of Indian economy, small and marginal farmers being major stakeholders (85% of the farming community)^1^. The heterogeneity of these farmers in terms of agroecology and resource endowments calls for the careful targeting towards the transfer of appropriate technology. Identification and characterization of farming systems may simplify the huge diversity of farm types in complex agroecosystems, which is of critical importance for precise technological intervention and informed policy support^2^. The adoption of new technologies in agriculture plays a pivotal role in building sustainable and resilient food systems^3^. After Green Revolution era, there had been a shift in cropping practices of farmers of Indo-Gangetic Plains (IGP), the area under cereal crops especially rice and wheat double cropping has shown a substantial increase^4^. This brought forth several edaphic, environmental, and social implications, several problems have cropped up in the region with the dominance of cereal-cereal based system for the last six decades ultimately threatening the sustainability of the system due to lack of diversity as well as economic stability of the farmer. Now a days, income from crops is the major source of income for farmers. To ensure livelihood security and risk coverage farmers need to have multiple sources of income irrespective the percent contribution to income. Especially for small and marginal farmers integrated farming system (IFS) seem to be promising solution for economic stability^5^. Higher adoption may lead to enhanced yield, increased resource use efficiency creating opportunity for production-led impact on economy and rural livelihoods. The inherent variability often influences farmers’ response to various technologies that aim at improving farm productivity, profitability and natural resource management^6,7^. However, there are numerous unfortunate examples of technologies with great potential that have not been accepted by the farming community, especially the smallholders of the developing countries. The reason being, quite often, these technologies do not fit well into heterogeneous smallholder systems, requiring specific technological solutions. Extension offered blanket recommendations for wide geographical areas that was largely used as a deterministic ‘dart gun’^8^, i.e. ‘take the technology and transfer it to farmers’. Thus, study of farm heterogeneity is of paramount interest for effective recommendation of technologies. Typologies aid in realistic evaluation of constraints and opportunities faced by farmers and helps forwarding appropriate technological solutions, policy interventions^9–11^, as well as comprehensive environmental assessment^12^. Moreover, it helps in understanding the factors that explain the adoption and/or rejection of new technologies^13,14^. This exercise can be made by the researchers, end-users and policy makers through developing farm typologies as a major tool for dealing farming system heterogeneity^15^. Researchers have examined factors such as farm resources like cash and labor^16^, infrastructure such as marketing agency and markets^17^, management practices^18^ and technological level^19^ and few others have used a string of factors together to explain the heterogeneity of farming systems^20–23^. Farm typologies have been used to study climate change adaptations^24^, resource use efficiency^25^, water use efficiency^26^, integrated pest management^27^ and may sometimes be crop specific in nature^28,29^. Most of the farm typologies studies have focused on socio-economic and agroecological factors for classification of farms especially in small-scale studies, for classifying farms^30,31^. The selection of variables that outline farm typologies should be decided by the objective of the research. This study assumes that classification of farms based on contribution of farm enterprises, together with other related non-economic factors, will provide meaningful insights into the farm type identification and planning targeted technology intervention for improved farm income.

In this study, we explored homogeneity in the farming system along an agroecological gradient of the IGP and integrate this understanding in exploring possible interventions for improving farm income.

## Materials and method

The research was carried out through the on-farm research centers of Indo-Gangetic plains of India under the aegis of ICAR-Indian Institute of Farming System Research (IIFSR), Modipuram, Meerut.

### Study area: location and farm household survey

**Figure 1:**
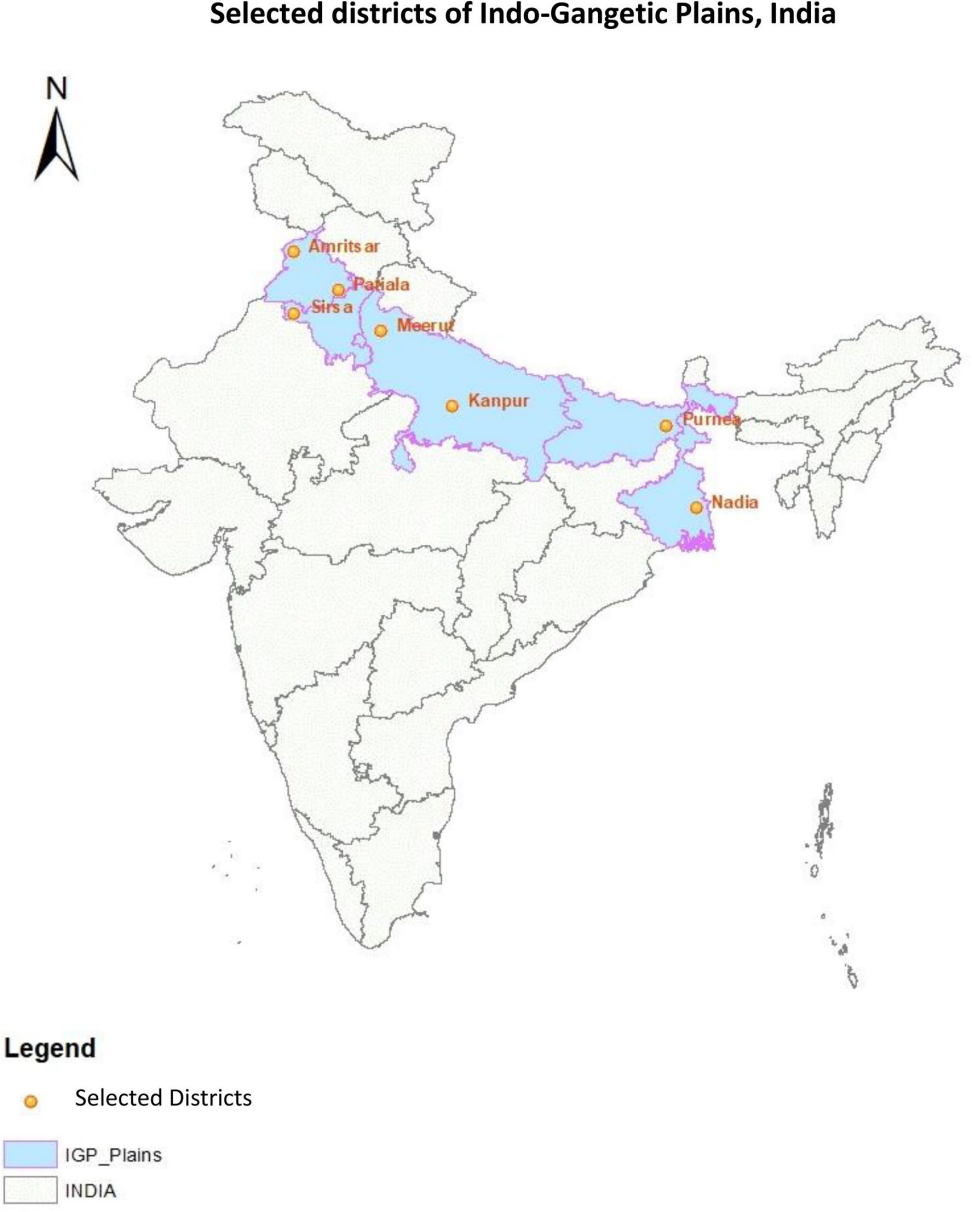
Map of study locale with selected districts of Indo-Gangetic Plains of India.

The survey for typology construction was carried out in 7 districts from 5 states viz. Amritsar and Patiala (Punjab), Sirsa (Haryana), Meerut, Kanpur (Uttar Pradesh), Purnia (Bihar) and Nadia (West Bengal) covering agro-ecological gradients of IGP (Fig. 1) by collecting information on structural and functional characteristics of farm and farming systems. The survey was implemented by collaborating centers of on-farm research program of IIFSR in their respective district. From each district total six villages were selected as a representative farming system of the locale. From each village six farm households were chosen randomly. Thus, thirty-six farm households (Farm HHs) were identified from each district and finally a total of 252 farm households were taken under consideration from IGP for the study. The survey questionnaire, comprising of 23 variables, approved under All India Coordinated Research Programme on Integrated Farming Systems (AICRP-IFS) was used to capture structural characteristics, cropping system, livestock related and income related characteristic (Table 1).

**Table 1:** Summary statistics for variables used for categorizing farm households.

Variable (n = 233)	Code	Unit	Formula	Mean
**Structural characteristics**				
Farmers id	id	ID	ID	–
District	district	Name	Name	–
Family size	familysize	Number	Number	4.5
Household head age	age	Year	Year	45.7
Family labour	onfarmlabour	Number	Number of persons working on farm	1.5(1–2)
Land owned	ownland	Ha	hectare	1.0
Land on rental basis	rented	Ha	hectare	0.0
^#^Land holding	totalarea	Ha	hectare	1.0
**Cropping system**				
^#^Area with cereals	cerealintensity	%	% cropped area with cereal	130.1
^#^Area under fodders	fodderintensity	%	% cropped area with fodder	8.2
^#^Area under cash crops	cashintensity	%	% cropped area with cash crops, oilseed, pulses, fiber	35.5
Area under other crops	othercrops	%	% cropped area with other crops flowers, orchards, vegetables etc	6.5
**Livestock related**				
^#^Total livestock units	tlu	Number	cattle-0.7 sheep- 0.1 goats- 0.1 pigs- 0.2 chicken- 0.01 ^36^	0.2
Total number of local cattle	localcattle	Number	Number	1.5
Total number of improved bred cattle	improvedbreed	Number	Number	0.6
Total livestock	livestock	Number	Number	2.1
^#^Total number of small ruminants	smallrumi	Number	Number of goat and sheep	0.5
Total number of small animals	animalsmall	Number	Number of poultry birds, pigs etc	0.2
Milk production per animal	milkperanimal	l/year	l/year	959.3
Total milk production	milk	Litres		2241.7
**Income related**				
^#^Income from crops	cropincome	%	% of total income	70.7
Income from livestock	incomelivestock	%	% of total income	25.3
Income from other sources	Others	%	% of total income	4.0
Mechanization	mechanization	Number	0-Animal Power, 1-Owned, 2-hired, 3-Mixed (Owned + Hired + Animal Power)	–

^#^Variables selected for PCA after correlation analysis.

The survey was performed in accordance with their relevant guidelines and regulations approved by the technical programme review committee of AICRP on IFS headed by Programme Coordinator and funding agency. We adhered to the Code of Ethics of the International Sociological Association (ISA) for the formulation and execution of the questionnaire. The questionnaire was also approved by the institutional committee at ICAR-IIFSR and pre-tested in the field before the final collection of data. Since the survey was interview-based with humans, before conducting the survey, we informed the participant about the purpose and the utilization of the survey, informed consent was obtained from each of the participants. The surveyed data was subjected to principal component analysis (PCA) and cluster analysis (CA) for typology construction. The technological interventions were carried out at selected farm households with technology and input support.

### Typology construction

#### Selection of variables

The diversity of farm households (HH) in IGP was explored for typology construction^32^. For this purpose, the structural (structural characteristics and livestock related) and functional (cropping system and income related) variables were computed (Table 1). The district wise means of studied variables are given in supplementary material (Annexure I). To avoid the effects of collinearity, the 23 variables were then subjected to correlation analysis and the variables which were significantly correlated were identified. From the inter-related variables, the variables which explains more diversity of data were selected. To avoid distortions in the statistical analysis, the dataset was carefully examined by evaluating missing data and identifying potential outliers. Boxplots were used to detect outliers which were deleted at the risk of improving the multivariate analysis while limiting it generalize ability to the entire population. Out of 252 farm sample households, 233 households were retained for statistical data analysis (i.e. 19 farm households were identified as outliers or containing incomplete data). Based on correlation analysis out of 23 variables 7 variables were chosen for further analysis (Table 1).

#### Multivariate ordination analysis

Two multivariate statistical techniques were employed sequentially for generating a typology of the surveyed farm households: Principal Component Analysis (PCA) to reduce the dataset into non-correlated components followed by Hierarchical Cluster Analysis (CA) for partitioning the PCA output into clusters. The approach has been used in many studies to categorize farming systems^33^. All analyses were executed in R (version 3.1.0) with the ade4 package (version 1.6-2, available online at: http://pbil.univ-lyon1.fr/ADE-4/) and the cluster package (version 1.15.2).

#### Principal component analysis (PCA)

PCA was applied to reduce the multivariate farm HH data set to non- correlated PC’s using ade4 package. The decision of how many principal components (PC’s) to keep was made based on three criteria: (1) according to Kaiser’s criterion, all PC’s exceeding an eigen value of 1.00 were initially retained (2) scree plot test and minimum cumulative percentage of variance chosen. The final criterion, that of (3) interpretability, was used to assess the conceptual meaning of the PCs in terms of the hypothesis under evaluation.

#### Cluster analysis (CA)

The PCA output in the form of a reduced dataset based on the retained PC’s was subjected to CA. A two-step approach was followed: first, a hierarchical clustering using Ward’s method^34^ and the maximum average silhouette^35^ were employed to define the number of groups or farm types in present case. Ward’s method results in a range of cluster solutions, where each observation starts out as its own cluster and is successively joined by similar observations/clusters until only a single cluster remains. This agglomerative nesting process is represented by a dendrogram and the decision to cut dendrogram was made by searching for maximum average silhouette width of different k- means clustering (method used for splitting dataset into set of k groups) with varying cluster numbers^35^.

After selection of number of clusters to be retained Kruskal–Wallis test was undertaken to determine the significance difference among variables in different farm types. In addition, to assess the mechanization status of different farm types, association plot was developed using *package vcd* to compare the scenario of mechanization among different type which will help in planning targeted interventions.

#### Farm type interpretation and analysis and district wise distribution

To characterize the final set of clusters, each cluster was examined in terms of their inherent structure (i.e. the mean value of each variable for each cluster) and were named accordingly. For assessment of district wise distribution of farm types, the proportion of farm types in all districts was computed and the results were presented in a hierarchical tree structured map using excel. This would help to identify which farm type prevails in that district and thus to formulate interventions for that specific type in the respective areas. Additionally, district wise mechanization status of the identified farm types was also studied, by identifying significant associations between types and mechanization level through chi-square test and its Pearson’s residuals visualization.

### Identifying constraints and possible interventions

Major constraints in farming system across the chosen locations based on the household survey data were identified. Afterwards consultation with survey and non- survey participants and the concerned local expert potential interventions to address the identified constrains were planned and executed to record the change in net returns obtained by farm HHs with tailormade interventions. The inputs required for interventions were provided to the farmers funded by IIFSR, Modipuram, Meerut.

## Results

### Farming system characterization

#### Principal component analysis

**Figure 2:**
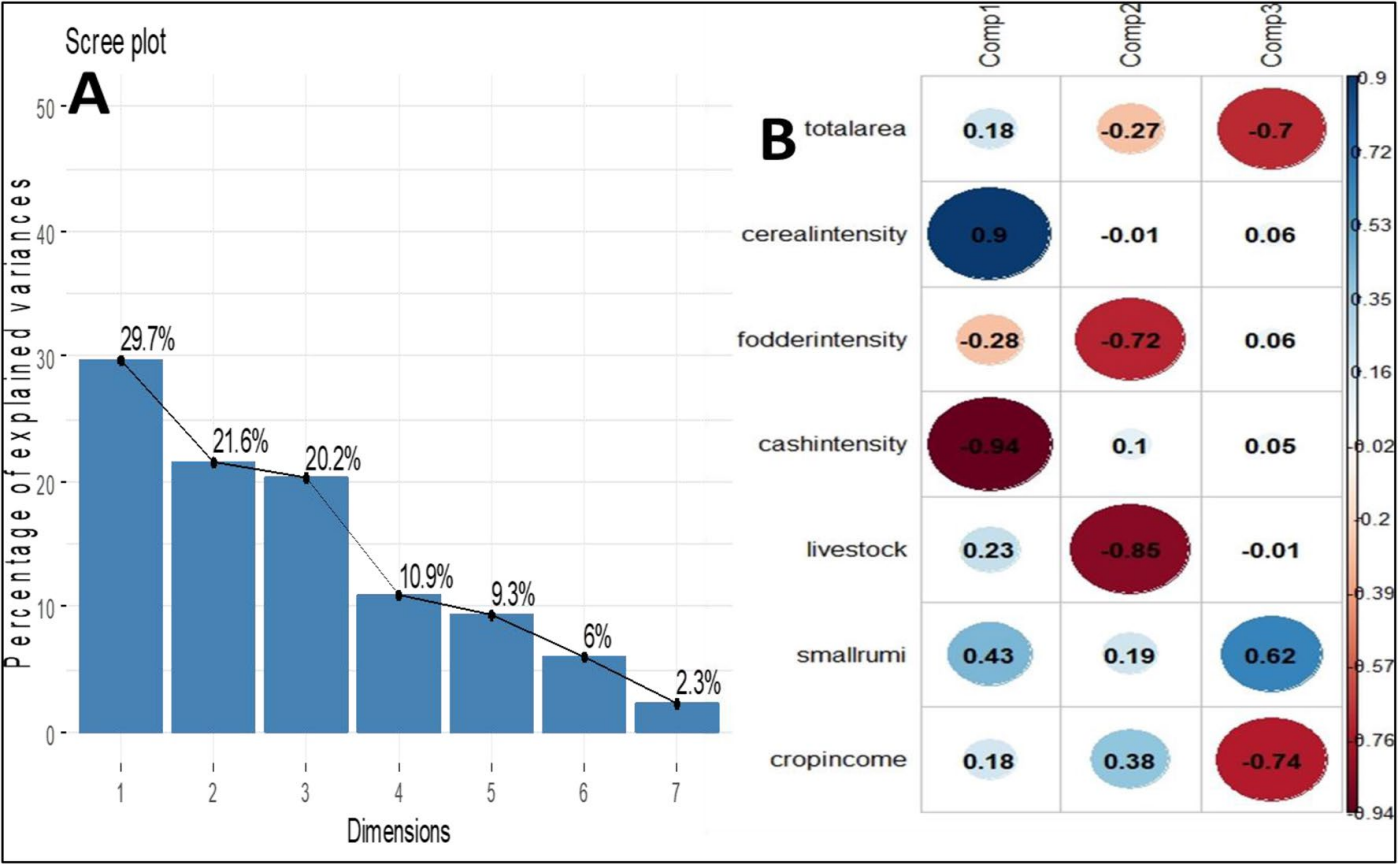
PCA result output: (**A**) Scree plot (**B**) Correlation plot of PCs with variables.

As per analysis of the HH survey data of 233 farmers, 23 variables were measured (Table 1) and results stated that, farmers had an average 1 hectare land holding, higher number of local cattle (1–2 numbers) in comparison to improved cattle breeds (0–1 numbers) with an average total milk production of 2241.7 litre per year. The farmers have higher proportion of income from crops (70.7%), 25.3% from livestock component and very less from other sources (4.0%). After correlation studies of the survey data, 7 variables were chosen for principal component analysis (PCA). PCA resulted in extraction of seven principal components, out of which 3 principal components were retained with eigenvalue more than one, explaining a total of 71.5% variance (Fig. 2A). Correlation plot (Fig. 2B) presents the loading of different variable on the principal components and the variables were related to cropping activities (diversity and intensity), relative importance of farming enterprises comprising of crop and livestock in income generation, livestock number (Fig. 2B). Component 1 explained 29.7% of variance and showed correlation with per cent area under fodder crops (fodderintensity = − 0.28), small ruminants (smallrumi = 0.43) but the discriminating variables were percent of land area under cereals (cerealintensity = 0.90) and cash crops (cashintensity = − 0.94). Negative correlations in PCA don’t cause any concern^37^. Component 2 explained 21.6% variance showed correlation with total land area (totalarea = − 0.27), percent crop income (cropincome = 0.38) but the component could be discriminated on the basis of area under fodder crops (fodderintensity = − 0.72) and livestock number (livestock = − 0.85). These two components together explained 51.3% of variance. Component 3 explained loadings of total cultivable area (own as well as leased) available with farmer (totalarea = 0.70), number of small ruminants (smallrumi = 0.62) and proportion of total income generated from crops (cropincome = − 0.74). Thus, this component represented the assets/income related components explaining 20.2% of variance in data.

#### Cluster analysis

**Figure 3:**
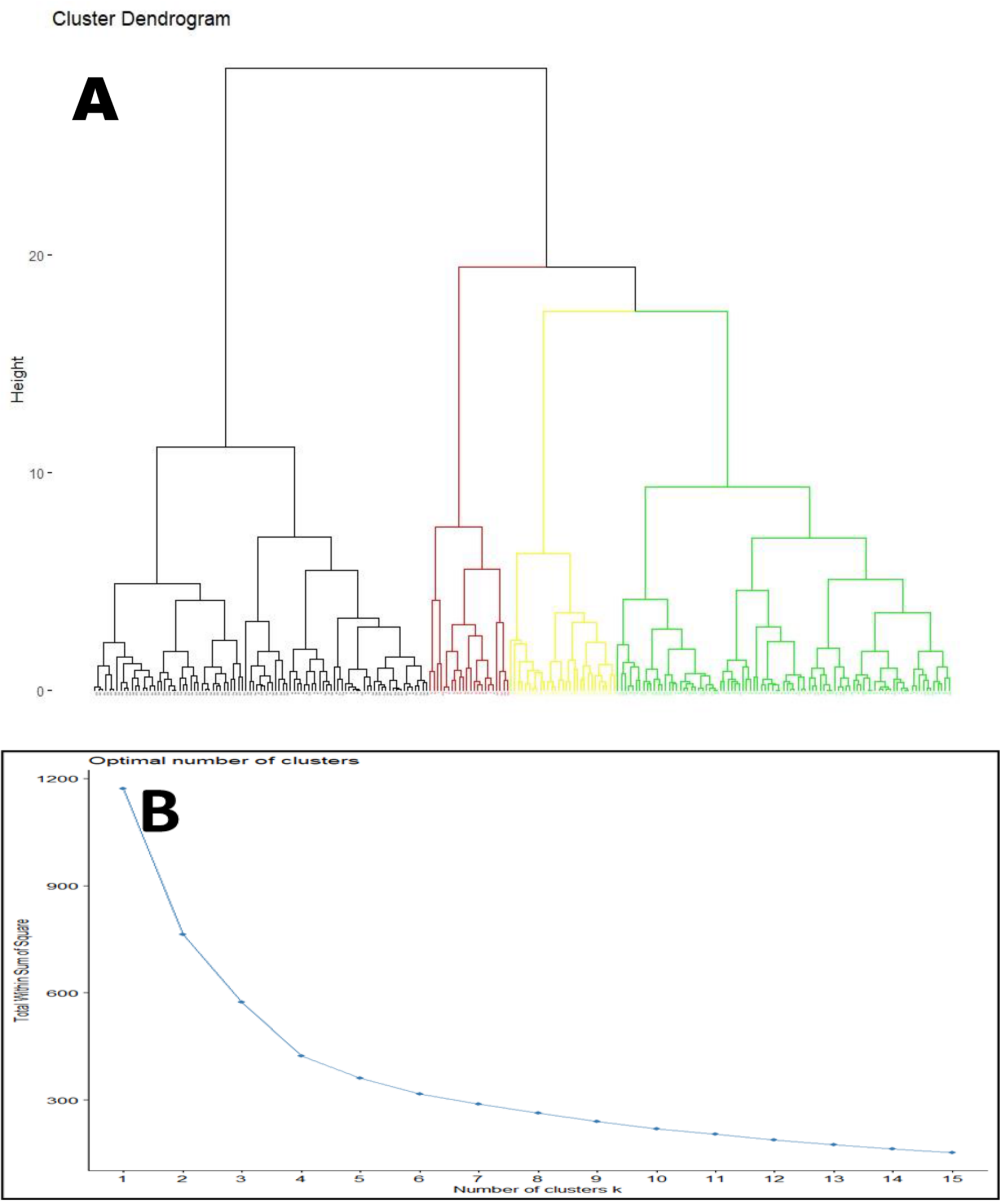
(**A**) Dendrogram and (**B**) Scree plot to choose optimal number of clusters.

The three principal components generated for the 233 farmer HH were used as input data for cluster analysis. Hierarchical clustering indicated 4 cluster cut off points grouped by structural and functional characteristics of the farm such as land and livestock resources as well as their main farming activities and income generated characteristics. The dendrogram was generated from agglomerative hierarchical clustering, it suggested 4 clusters and the scree plot also supported 4 clusters (Fig. 3).

#### Farm structure and function

The identified farm HH types were characterized based on magnitude of crop, livestock and income related characteristics (Table 2). Land area, cropping system, livestock ownership and diversity, source of income proved to be the clustering factors as evident from the correlation of PCs with the selected variables (Figs. 2B and 5A–C). Previous studies have reported, the effect of land and livestock ownership and cropping practice on clustering^32^. Thus, deep understanding of how these variables were represented in each type and their impact on decision making play crucial role in tailoring the interventions. Highlighting characteristics variables which defined the clusters are discussed below:

**Table 2:** Crop, livestock and income related characteristics of households under different farm types.

	Unit	Type 1	Type 2	Type 3	Type 4
**Structural characteristics**					
Family Size	Number	4.2^a^ (1.2)	4.3^a^ (0.7)	4.9^a^ (1.9)	4.6^a^ (1.3)
Household head age	Year	44.8^a^ (11)	42.2^a^ (10.3)	48.4^a^ (12.3)	43.1^a^ (10.5)
Family labour**	Number	1-2^ab^ (0.7)	1^b^ (0.4)	2^a^ (0.8)	1-2^b^ (0.7)
Land owned**	Ha	1.2^a^ (0.6)	0.9^ab^ (0.2)	0.8^bc^ (0.5)	0.6^c^ (0.2)
Land taken on rental basis	Ha	0.0^a^ (0.1)	0.1^a^ (0.4)	0.1^a^ (0.4)	0.0^a^ (0.0)
Land holding**	Ha	1.2^a^ (0.5)	1.1^a^ (0.5)	0.9^b^ (0.6)	0.6^b^ (0.2)
**Cropping system (%)**					
Area with cereals**	%	167.6^a^ (26)	150.6^b^ (33.6)	73.1^c^ (29.3)	176.2^a^ (26.7)
Area under fodders**	%	2.2^c^ (8.5)	36.9^a^ (25.2)	9.8^b^ (11.2)	0.9^c^ (4.6)
Area under cash crops**	%	7.2^b^ (15.1)	9.7^b^ (20.6)	81.3^a^ (22)	1.3^b^ (7)
Area under other crops**	%	1.8^c^ (7.4)	1.4^c^ (6.7)	10.7^b^ (21.2)	12.3^a^ (15.3)
**Livestock related**					
Total number of livestock**	Number	0.2^b^ (0.6)	0.4^a^ (0.2)	0.1^c^ (0.1)	0.5^a^ (0.6)
Total number of local cattle**	Number	1.6^b^ (1)	3^a^ (1.6)	1.1^c^ (0.8)	1.4^bc^ (0.9)
Total number of improved bred cattle**	Number	0.6^b^ (0.7)	1.8^a^ (1.3)	0.5^b^ (0.8)	0.0^c^ (0.0)
Total livestock unit**	Number	2-3^b^ (1)	4-5^a^ (1.4)	1-2^c^ (0.8)	1-2^c^ (0.9)
Total number of small ruminants*	Number	0.1^bc^ (0.4)	0.5^b^ (1.4)	0.0^c^ (0)	3.6^a^ (2)
Total number of small animals	Number	0.3^a^ (1.5)	0.0^a^ (0.0)	0.1^a^ (0.6)	0.9^a^ (4.6)
Milk production per animal**	l/ha	881^a^ (554)	1145^a^ (452)	1097^a^ (626)	547^b^ (326)
Total milk production**	Litres	1981^b^ (1510)	5458^a^ (2931)	1703^b^ (1075)	938^c^ (530)
**Income related**					
Income from crops**	%	78.7^a^ (17.3)	55.9^c^ (17.2)	68.5^b^ (18.7)	59.8^bc^ (20.4)
Income from livestock**	%	18.4^c^ (14)	39.9^a^ (13.1)	27.1^b^ (15.9)	28.7^b^ (18.2)
Income from other sources**	%	1.6^b^ (7.4)	0.0^b^ (0.0)	4.1^b^ (10.5)	12.7^a^ (21.1)

As per Kruskal-Wallis test, variables denoted with * differ significantly with p < 0.05 and with ** differ very significant with p < 0.001.

Data figures with different letter (a,b,c) are significantly different among means of 4 farm HH types.

Figures in the parenthesis are standard deviation of mean.

**Figure 4:**
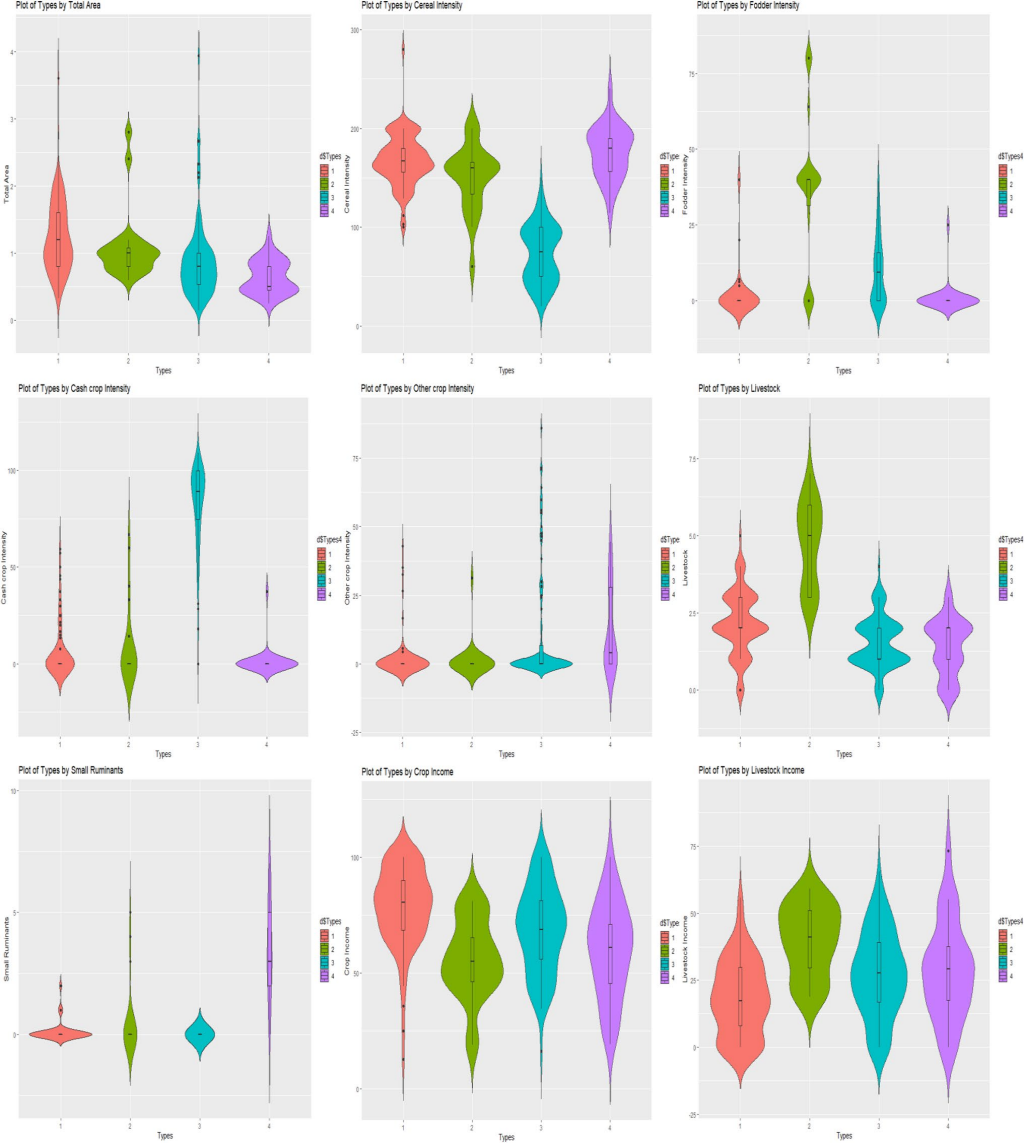
Violin plots showing results of scenario analysis of different variables in farm types.

*Structural Characteristics* The size of land holding is the key component which plays a vital role in farming practices and the identified farm HH types had significantly different total land holding size. Violin plot of different farm HH types with total area showed that Type 1 and Type 2 farm HH had similar land holding size which was significantly higher in comparison to Type 3 and Type 4 farm HH. Also the shape of the violin plot showed that the land holding in Type 2 farm HH is more concentrated around mean (1.1 ha) however, in Type 1 it had more variability (Table 2; Fig. 4A).

*Cropping system* The Type 1 and Type 4 farm HH were positively skewed with cereal crops as they were having significantly higher cereal intensity than Type 2 and 3 farm HH. Maximum households in type 1 and 4 had cereal intensity higher than 100 per cent (Fig. 4B). On the other hand, type 2 farm HH had significantly higher fodder intensity (36.9%) (Table 2) as well as median value (Fig. 4C) in comparison to other types. Type 3 farm HH had more diversification in their cropping practices, they had higher cash crop intensity (Fig. 4D) over cereals. They also grow fodder and other crops. Area under other crops were significantly higher in type 4 (12.3%) followed by Type 3 (10.7%) which were significantly higher than type 1 and 2 (Fig. 4E).

*Livestock related characteristics* Type 2 farm HH had significantly higher number of livestock (4–5 numbers) (Table 2) than other HH, as depicted in the violin plot (Fig. 4F). Negligible households in type 1, 3 and 4 had more than 5 livestock unit. Type 4 farm HH had significantly higher number of small ruminants (3–4 numbers) (Fig. 4G), whereas type 1 and type 3 had negligible ownership for the same (Table2).

*Income related characteristics* Income from crops (Fig. 4H), livestock (Fig. 4I) and other sources varied significantly for different farm HH type (Table 2). Type 1 had significantly higher income from crops (78.7%) followed by Type 3 (68.5%), Type 4 (59.8%) and Type 2 (55.9%). However, different trend was observed w.r.t income from livestock, Type 2 farm HH had significantly higher share (39.9%) from livestock component, followed by Type4 (28.7%), Type 3 (27.1%) and least in Type 1(18.4%). Type 4 were engaged in off-farm work thus getting income from other sources (12.7%), as this type farm HH had possession of minimum land holding than other types, thus, are inclined to other activities to ensure livelihood security. However, Type 2 farm HH had negligible share from other sources which might be due to their preference towards livestock as they have considerably higher contribution from livestock component in comparison to other types.

On perusal of structural and functional characteristics in accordance with the identified clusters variables land holding, cereal intensity, fodder intensity, cash crop intensity, total livestock, income from livestock and other sources were significantly different for different types and proved to be discriminating variables for cluster identification after PCA. The resulting types were named as per their respective possessions:

- Type 1. *Small Farm households with cereal-based cropping system and subsistence livestock* (39% of the sampled farms)

This type was differentiated from the other types due to the strong discriminating power for variables related land holding (1.2 ha) having second largest area under cereal crops (167.6% cereal intensity) among other types. This type relied heavily on the sale of crop products as 78.7% share of income is obtained by selling crop produce. Conversely, the percentages of livestock sales were the lowest among all farm types (18.3% of the share in income from livestock component).

- Type 2. *Small Farm households with diversified cropping system dominated by cereal and fodder crops with only cattle herd* (9% of the sampled farms)

Type 2 was the smallest cluster, characterized by small farm HH (1.1 ha) diversified in cropping system having 150.6% cereal intensity, largest fodder intensity (36.9%) among all other types. This type possessed livestock unit consisting entirely of cattle. The crop and livestock sources had 55.9% and 39.9% ratio respectively in total income of the farm HH.

- Type 3. *Marginal Farm household with diversified cropping system dominated by cash crop and herd comprising of only cattle* (39% of the sampled farms)

**Figure 5:**
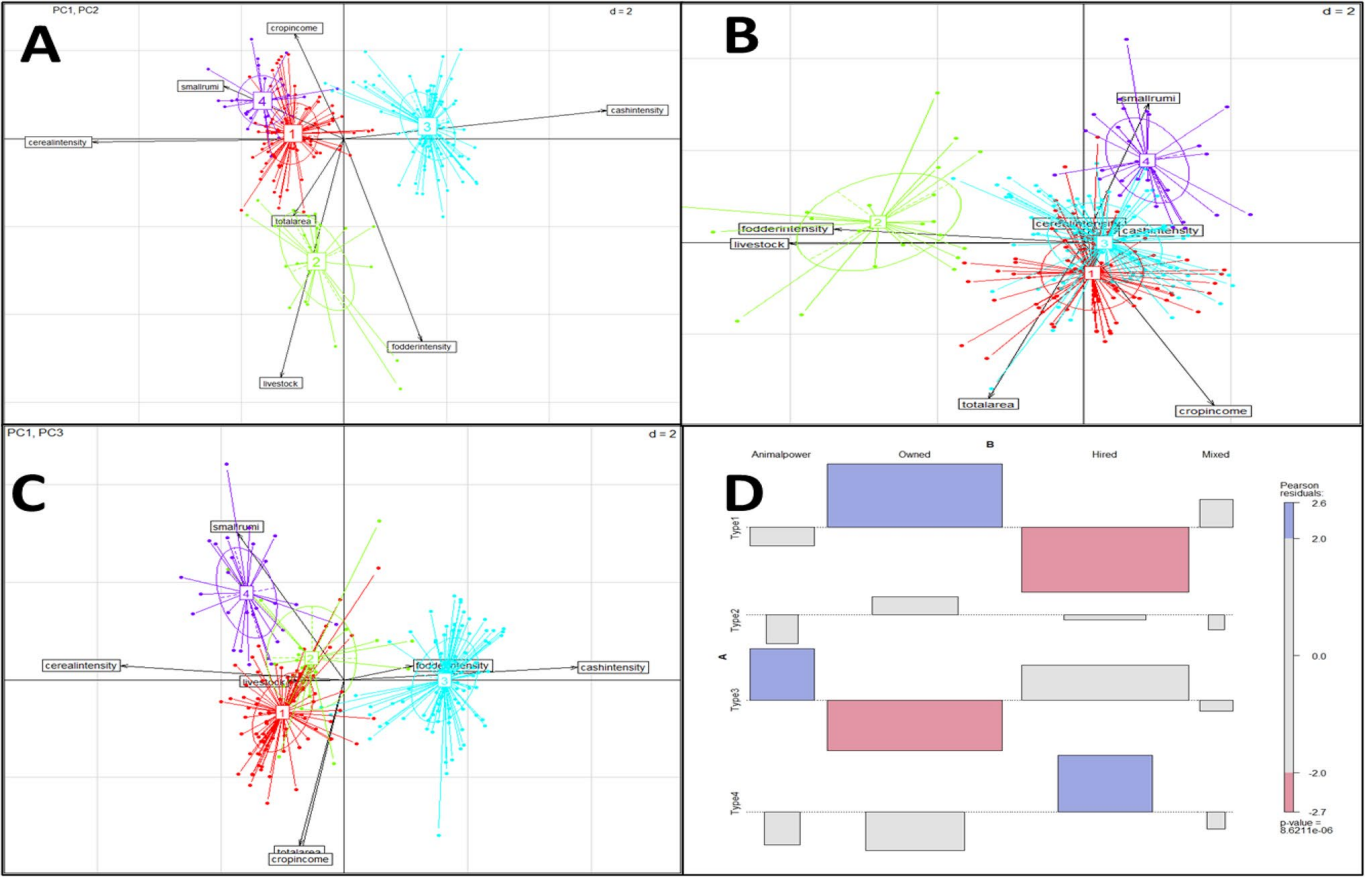
(**A**–**C**) Spatial distribution of different farm types. (**D**) Pearson residuals visualization after chi-squared test of mechanization status *vs* different farm types (Blue rectangles show significantly positive associations, and pink rectangles depict significantly negative associations, gray rectangles show non-significant associations).

Type 3 was characterized by the marginal farm HH (average of 0.9 ha), with diversified cropping system and largest area under cash crops with 81.3% cash crop intensity, 73.1% cereal intensity, 9.8% fodder intensity. Livestock component consisted only of cattle which is for subsistence (1–2 local cattle with milk production 1703 litre/year and is also used as draught (Fig. 5D) contributing 27.1% share to income.

- Type 4. *Marginal Farm household with diversified cropping system dominated by cereal crops and herd dominated by small ruminants* (12% of the sampled farms)

For Type 4, the main distinguishing features included herd composition and cereal intensity. The herd consisted mainly of small ruminants (on average 1–2 cattle, 3–4 small ruminants). With 0.6 ha of average land holding, this group cultivated the largest area under cereals with 176.2% cereal intensity. The farm HH in this group had highest contribution from other sources of income (12.7%) in comparison to other groups.

### Distribution of different types of farm households in different districts

**Figure 6:**
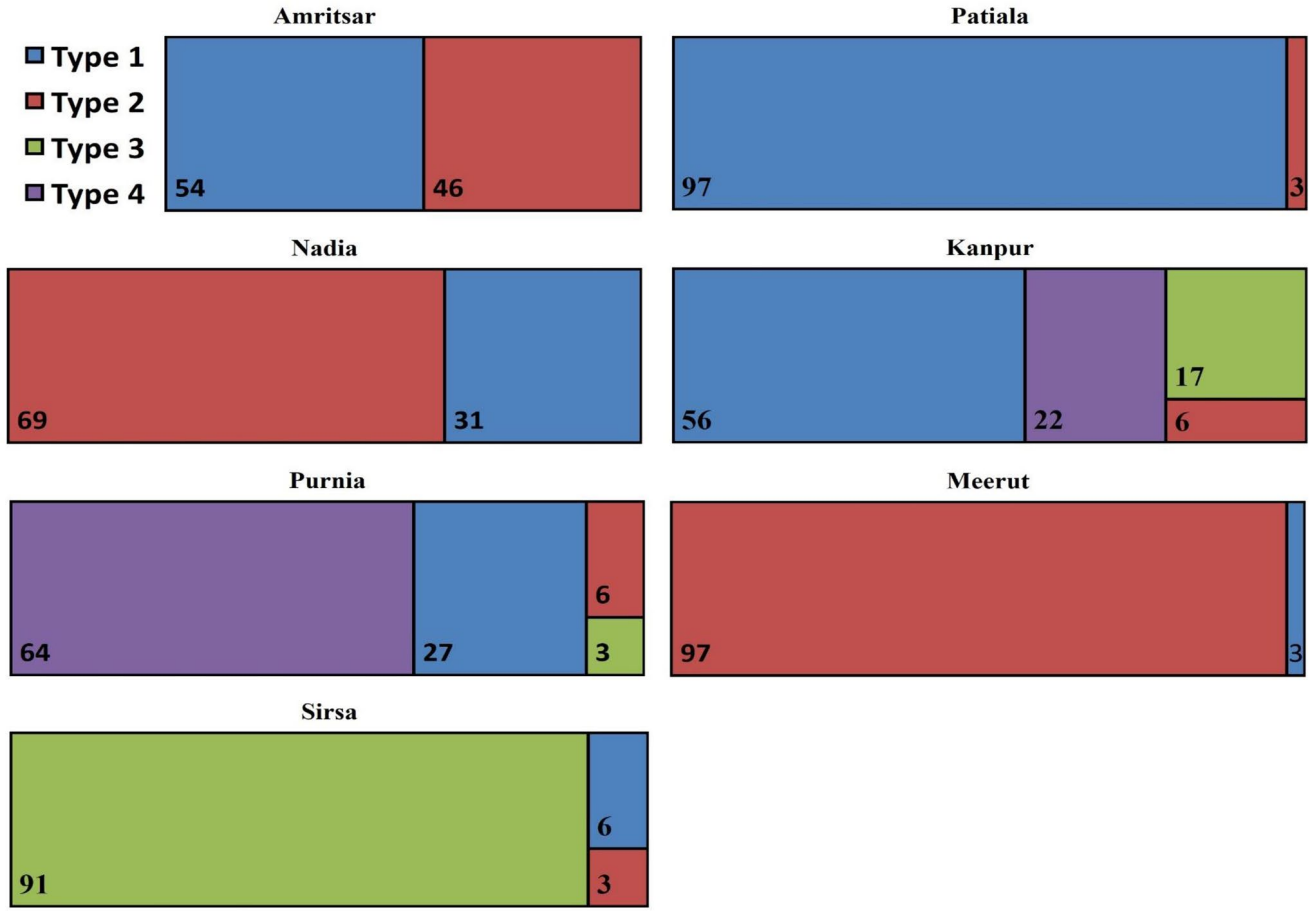
Distribution of farm types (%) in different districts.

Approximately 39% of the farm HH belonged to type 1 and type 3 each and both being crop type farmers, type 1 being the cereal farmer whereas type 3 being the cash crop farmers. Type 2 (9%) and type 4 (12%) represented the mixed famers having crop + livestock contribution in their income. Different farm HH types were not uniformly distributed among selected districts. In Amritsar district, there was almost equal distribution of Type 1 and Type 2 farm HH i.e. 54 and 46% respectively (Fig. 6). Patiala district had farm households that predominantly belonged to type 1 which are the cereal intensive farm HH (97%). In Nadia there was predominance of Type 3 farm HH (69%) rest being type 1 farm HH. District Purnia and Kanpur had representation from all 4 farm HH types, although Kanpur had dominance of cereal intensive farm HH whereas Purnia was dominated by marginal diversified farm HH. In Meerut, the prevalent farm types were type 3 (97%) and rest is type 1, i.e. district was dominated by crop growers. In Sirsa, there was dominance of type 3 farm types and small proportion of type 1 and type 2. The National Agricultural Research Project Zone (NARP), its sub-region and their characteristics of selected districts are given in supplementary material (Annexure II).

### Mechanization status of different farm types

**Figure 7:**
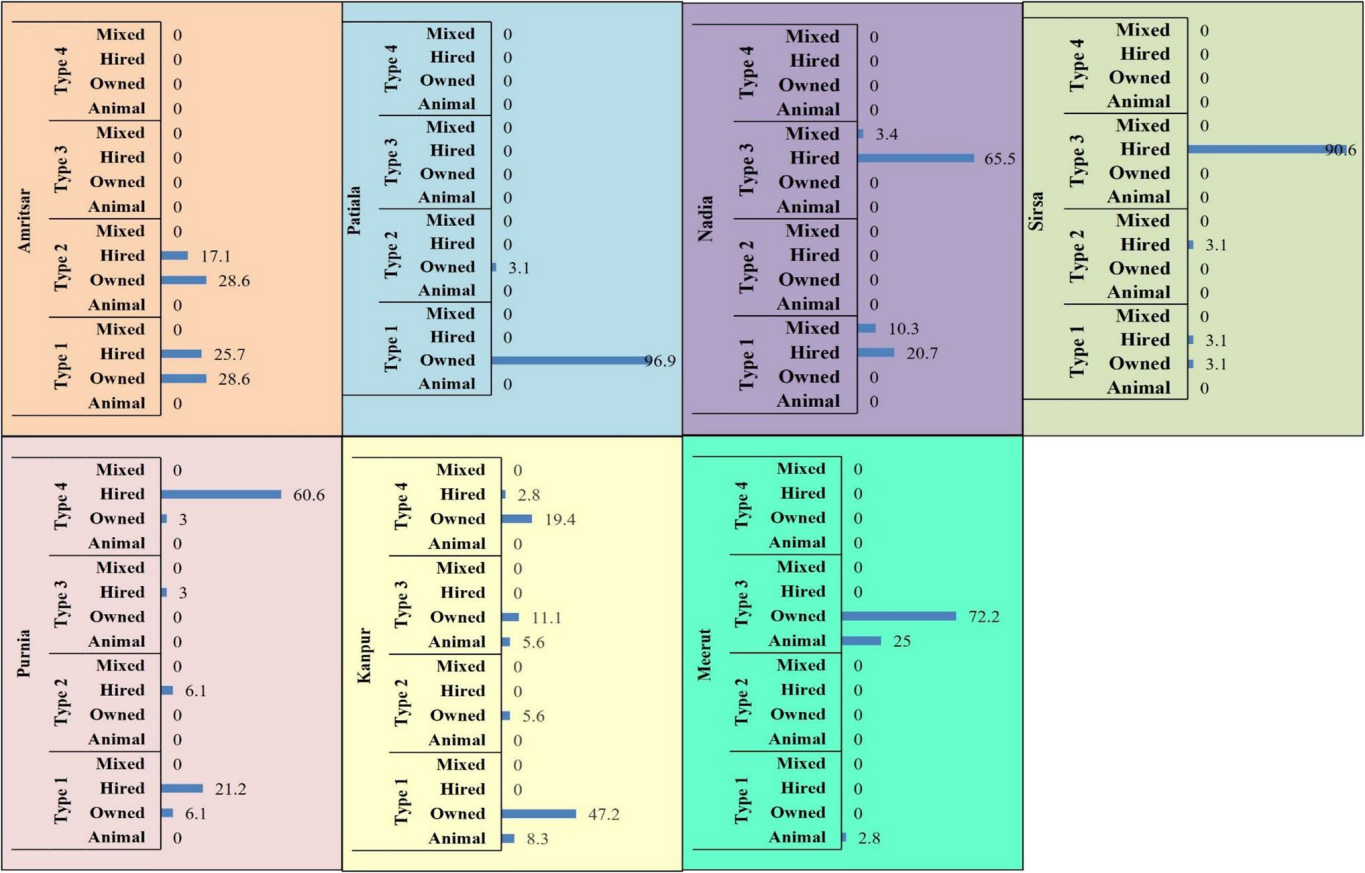
Status of mechanization (%) in different districts w.r.t types. Animal–Animal Power, Mixed − (Owned + Hired + Animal Power).

Mechanization in farming practices was also studied in IGP for evaluating the access of the farm HH types to machinery to understand the choice of farming practices and to further draft the interventions. There was a significant positive association of Type 1 with owned machinery and negative association with hired machinery as they were the cereal growers, type 2 didn’t exhibit any significant association with mechanization (Fig. 5D). Type 3 had shown a positive association with animal power and negative association with owned machinery. Type 4 was positively associated with hired machinery. Mechanization % in different districts is presented in in Fig. 7. Districts Amritsar, Patiala, Nadia, Purnia, Sirsa were among the mechanized districts, where the different farm types were either owning or hiring machinery (Table 4). District Patiala had maximum farm HH having own machinery (Type 1–96.9% and Type 2–3.1%). The Amritsar, Sirsa, Nadia and Purnia districts have majority of farm HH using own or hired machinery, whereas, Kanpur had Type 1 farm HH (8.3%) and Type 3 farm HH (5.6%) using draught animals for farming. Similarly, Meerut had significant proportion (25%) of Type 3 farm HH using animal power for cultivation. The status of mechanization also depended upon the crop grown by farm HH. In the districts with type 1 and type 4 farm HH (except Meerut where they had animal power) having higher cereal intensity, with mechanized farms either hired or owned.

### Identification of constraints and possible interventions

The farm HH under study were of diverse nature. Based on the resources endowed by them, agricultural practices followed, economic decisions made, etc., diversity existed among the sampled farm HH. There was a need to fully recognize that diversity and to identify the constraints experienced by each type and to develop the modules suitable to address those constraints. The constraints have been enlisted in the Table 3. For the crop component, the non-availability of quality seeds of high yielding variety was a very serious constraint as per farmers. Also, after a certain peak, the yield has been stagnated, that may be due to high weed infestation, or imbalanced nutrient application, etc. Due to migrating population, there had been a raise in labour cost availability had reduced. As per the farmers’ perception, due to persisting problems and unsteady markets especially for cash crops, agriculture had not been a remunerative system. Increased cost of cultivation due to overuse of agrochemicals, less residue recycling (as compost), lack of technical knowledge had also became a limiting factor for farming community.

**Table 3:** Constraints and problem identified (farm type wise) in studied districts falling under IGP (Based on survey).

Constraints	Farm types	Amritsar		Patiala		Nadia		Sirsa			Purnia				Kanpur				Meerut	
		1	2	1	2	1	2	1	2	3	1	2	3	4	1	2	3	4	1	2
**Crop component**	**Problem identified**																			
Low yield of crop/Yield Stagnation	Availability of high yielding improved variety seed	**√**	**√**	**√**		**√**	**√**	**√**	**√**		**√**			**√**					**√**	**√**
	High weed infestation	**√**		**√**		**√**	**√**					**√**	**√**							
	Crop damage by stray/wild animals																			
	Poor soil health	**√**	**√**	**√**						**√**										
	Imbalanced nutrient application	**√**	**√**	**√**	**√**	**√**	**√**			**√**	**√**		**√**	**√**	**√**			**√**	**√**	**√**
	Insect-Pest problem	**√**		**√**	**√**		**√**						**√**	**√**						
	Micro nutrient deficiency					**√**	**√**					**√**	**√**						**√**	
Low income	Low market price for vegetables and fruits				**√**															
	Lack of awareness of diversified crops fetching more price	**√**		**√**							**√**				**√**				**√**	
	Unstable price of cash crops										**√**									
	Increased cost of cultivation		**√**																	
	Low rate of residue recycling		**√**				**√**				**√**			**√**						
	High labour cost and low availability	**√**	**√**	**√**						**√**										
	Lack of technical knowledge about value addition of crop products	**√**	**√**				**√**					**√**	**√**	**√**						
**Animal component**																				
Low milk production	Mineral deficiency	**√**	**√**	**√**				**√**		**√**					**√**		**√**	**√**	**√**	**√**
	Poor health and Imbalance feeding			**√**	**√**			**√**	**√**	**√**					**√**	**√**	**√**		**√**	
	Sterility problem																		**√**	**√**
Fodder scarcity during lean period	Lack of knowledge on Silage and hay making			**√**		**√**						**√**		**√**					**√**	**√**
Low fish production	Improper rearing practice					**√**	**√**							**√**						
Low income	Low market value of milk	**√**		**√**						**√**										
	Lower market price for small ruminants																			
	Lack of scientific knowledge about other animal rearing (poultry, goat etc.)	**√**	**√**	**√**	**√**	**√**		**√**	**√**	**√**	**√**	**√**	**√**		**√**	**√**	**√**			
**Other income sources**																				
Low income high risk in agriculture	Lack of know how about allied enterprises	**√**	**√**	**√**	**√**	**√**	**√**	**√**	**√**	**√**	**√**	**√**	**√**		**√**	**√**	**√**		**√**	**√**
Low food self sufficiency	Lack of know-how on organic kitchen garden	**√**	**√**	**√**	**√**	**√**	**√**	**√**	**√**	**√**	**√**	**√**	**√**	**√**	**√**	**√**	**√**	**√**	**√**	**√**

For animal component, lower income was due to low milk production as a result of mineral deficiency, poor health and non-availability of good quality fodder during lean period. Also, the farm HH lack technical know how about rearing of livestock component other than cow and buffalo, they weren’t aware of the scientific approach in fishery, poultry and goat farming. The farm HHs, reported, low income and high risk in agriculture. They consider their dependency on crop and livestock as one of the key factors behind limping economic situation of agriculture dependent families. They felt the lack of information about the allied enterprises and also inability to proceed towards self-marketing, value addition of produce worsen the condition. Focus on cereal crops indirectly resulted in dependency on market for other food items.

After identification of the problems, a framework for possible low-cost interventions for representative farms (6 HHs from each type) was implemented (Table 4) and evaluated at farmers’ field based on identified constraints for farm types as well as availability of resources at farm HHs. No intervention involving drastic change in existing farming system was considered, rather refinement of existing system was carried out considering the risk bearing capacity and choice of the farmers, knowledge about the selected enterprise and available resource. The benchmark income was compared to the income obtained after technological intervention and results obtained in terms of net returns is presented in Table 4. For addressing low yield of field crops improved varieties along with improved insect pest management practices were introduced along with the technical know-how to include those in future also. Recommended fertilizer application for balanced nutrition in crops was also included for higher yield to contribute towards profitability. Further, to enhance the income from crop component, diversification with new crops (pulses, oilseed) fetching promising prices, intercropping in cash crops were introduced. For making agriculture more remunerative mere dependency on crop and dairy was not promising. Inclusion of improved small ruminants and backyard poultry besides improved technology for existing livestock were integrated for enhancing income from livestock component. To address the concern related to animal health and milk production interventions like inclusion of fodder crop for year-round fodder availability, mineral mixture supplementation, deworming of animals were considered. Nutritional kitchen gardening was incorporated in all farm types. For recycling of resources, vermicomposting was also included for utilizing the waste of one enterprise as input to the other enterprise.

**Table 4:** Effect of Farm type wise low-cost intervention on net returns.

Farm type	Prevailing system	Mean landholding of 6 Households (ha)	Benchmark Net returns (INR) (2018–19)	Low cost farming systems interventions	Net returns after intervention (2019–20) (INR)	% increase
Type 1 *(Small Farm households with cereal-based cropping system and subsistence livestock)*	Field crops (Cereal based) + dairy	1.56	149,928	Improved herbicides, seed treatment with Fungicides, Application of water at critical stages in wheat Nutritional kitchen gardening and summer moong for diversification Mineral supplements, Vaccination and De-worming in livestock and fodder crop	277,118	84.8
Type 2 (*Small Farm households with diversified cropping system dominated by cereal and fodder crops with only cattle herd)*	Crops (Diversified cropping system including fodder crop) + dairy + horticulture	1.12	104,370	High yielding variety of crops and berseem for year-round fodder production Vermicomposting Nutritional kitchen gardening and integrated pest management in orchards	203,221	94.7
Type 3 (*Marginal Farm household with diversified cropping system dominated by cash crop and herd comprising of only cattle*	Crop (Field crops + Cash crops + fodder) + dairy + horticulture	0.89	89,011	Improved seed and integrated pest management, intercropping in sugarcane Nutritional kitchen gardening Mineral mixture supplementation, deworming and fodder block for cattle Vegetables as intercrop in juvenile orchards	167,570	103.2
Type 4 (*Marginal Farm household with diversified cropping system dominated by cereal crops and herd dominated by small ruminants)*	Field crops (cereal based) + dairy + small ruminants/Fishery	0.72	61,100	Improved variety and recommended fertilizer application in crops. Diversification of cereal crops with pulses and oilseeds, fodder crops Mineral mixture + deworming, improved breed of small ruminant, feed management in fishery and proper stocking density, Integration of backyard poultry Nutritional kitchen gardening	120,978	98.0

There was significant improvement in net income all 4 farm HH types, among small farm households, type 1 farmers with cereal crop-based system and subsistence livestock showed 84.8% improvement in net income with use of improved crop production technology, inclusion of diversified crops and improved livestock raising interventions. While for type 2 farm HH, it consisted of cattle based diversified cropping system, thus for recycling the cattle waste was used as for vermicompost preparation and as diversification module improved fodder crop and horticulture crops were introduced which resulted in increase of 94.7% in net income over prevailing system. Similarly, Type 3 farm HH were marginal diversified farms with cash crops, so within limited availability of land, more crops were introduced as intercrops and for improving income from livestock, improved rearing practices were introduced, these interventions resulted in 103.2% enhancement in net income. Type 4 farm HH had limited availability of land, even though those farms had a diversity, they had more inclination towards cereal cultivation (Cereal intensity = 176.2%), thus more diversified crops (pulses, oilseeds, fodder crops) were introduced and for enhancing income from livestock backyard poultry was integrated. These interventions reported 98% increase in net over prevailing system.

## Discussion

Conventionally the farm HHs were classified mainly only on the basis of the size of land holding in possession, i.e. marginal, small, semi-medium, upper-medium, and large farmer^33^. In this study, the typologies are developed based on the possession of assets viz crop, livestock and decisions made by them related the crops and livestock rearing. Our analysis has clustered the farm HHs into four clusters based on structural characteristics, cropping system, livestock possessed, source of income and differentiate among different farm HHs. The farm HH have reported approximately 1–1.5 hectare average land holding or less. Similar finding have also been reported regarding the average land holding size declining over past years and has come down from 2.82 hectare in 1970–71 to 1.16 hectare in 2010–11^38^. Along with the shrinking holding size, as a result of green revolution in 1960s and economic liberalization in 1990s, the focus of farmers started shifting to few enterprises due to several factors like fluctuating prices of commodity, labour shortage during peak agriculture season, etc.^39^. These factors have imposed a severe impact on resource deprived farm HHs. Income enhancement in such cases is only possible by judicious integration of farm resources keeping in view the ecological conditions of the locality^40^. In present study, interventions consisting of improved crop cultivation practices, diversified crops, improved livestock rearing practices, waste recycling, inclusion of poultry reported increase in income ranging from 84.8 to 103.2 percent (Table 4). Economic benefit ranging from Rs 7880/ha to Rs 57,530/ha has also been reported by adopting different enterprise combinations of crop and dairy system with poultry, fishery, sheep and goat and horticulture^38^. It confirms that integrated farming system approach (IFS) proves to be beneficial when practiced according to ecological and socio-economic structure, choice of the farmers as well as resource availability of the farmers^41^. The adoption of integrated farming approach could generate per hectare additional income, depending on inclusion of kind and number of additional farm enterprises and their effective combination as reported by Ponnusamy and Gupta^42^. As evident from the analysed data, the farm HH’s having small land holding size will be more inclined towards other small animal components or off farm income sources. The farmers under study have either small or marginal land holding. With decreasing availability of agricultural land, typology derived targeted intervention approach in a systems approach is needed to be adopted for livelihood security. The interventions planned for different districts depending upon their resource allocation and market demands, will help to increase in the farm HH income. By adopting these interventions small and marginal farmers can proceed towards sustainability and economic viability of the agricultural production system (Table 4). It is difficult to sustain the farm family from crops income throughout the year, so regular cash flow is required which is only possible when the crop is combined with judicious combination of enterprises feasible in the environmental conditions of the area^43^. Kumar et al.^5^ also reported that when cropping system (Rice–wheat) was combined with other enterprises (Cropping + poultry + goat + mushroom) provided enhanced net return of 302% as compared to cropping systems alone. Recycling of residues/wastes also plays important role in sustainability of farming system^44^. Nutrient recycling enables self-sustainability of the system and reduce dependency on the external inputs viz, seed/ fertilizers etc. thus reducing the cost of cultivation which leads to enhanced profit. The integration of resources enable farm HH to reduce cost of production through recycling on-farm wastes and using by-products of one enterprise as input to other enterprises^40^ and thus minimizing the external inputs^45^. Kitchen gardening not only helps towards food self-sufficiency but also provides the balanced and rich nutrition to the farm family reducing cost of cultivation and increase profit from the same piece of land. To decrease cultivation cost and to increase income, multi- enterprise system of agriculture can play important role^46^. The integration of crops, livestock, fishery components that sustains food and nutritional security with regular and periodic income to farmers is vital^47^. The integrated approach encourages ecological intensification and aims to reduce use of anthropogenic inputs with enhanced ecosystem functioning^48^ like nutrient recycling, soil formation, soil fertility enhancement, and improving environmental performance^49^. Efficiently managed farming systems are expected to be less risky, as they benefit from enterprise synergies, product diversity, and ecological reliability^50^. Complementarities existing between components helps towards enhancing system output in holistic farming systems^48^.

Integration of resources had proven to be an excellent opportunity to enhance the yield per land area^45^ and to ensure food security as well as income generation to the rural poor^46^^.^^51^. Prototype farming systems developed based on characterization of the region comprising the different components like crop, dairy, fishery, horticulture and apiary in the study region was found to be sustainable and eco-friendly^52^. Another study conducted in Punjab indicated doubled productivity of crops + dairy and crops + dairy + poultry production systems in comparison to sole cropping^53^. Small ruminants (sheep and goat) have played a significant role in the agrarian economy^54,55^. Inclusion of goat farming is found suitable for landless and marginal group of farmers for productivity enhancement and improving farmers’ income^56^. The targeted intervention based on farm typology if done in integrated manner could provides risk coverage to farm HH against fluctuations in prices and climatic conditions as farmer can tactically adjust the allocation of input (land, water) across and between enterprises accordingly and choose cropping systems and enterprise based on objectives like profitability, meeting household requirement etc.

## Conclusion

In current study we have presented statistical methodology to characterize farm households and demonstrated the effect of targeted interventions. The heterogeneity in socio-economic and cropping practices have formed basis for developing homogeneous types. In our results, four Farm household types were identified among the seven districts of IGP and the interventions were planned considering the variability existing across different farm types. The results suggest that instead of the blanket application for whole area, recommendations to enhance farmers income should be case specific. Interventions planned through identification of farm types approach provided ray of hope to enhances the net return, to reduce the cost of production and to increase farm income in a holistic manner which could be upscaled further to farm types for reaping the benefits especially by resource constraint farmers.

The key contribution of this study is that it establishes need for socio-economic characteristic based farm types and also explains the benefit of typology based interventions on returns obtained by farm households. Further, district level intervention planning could be implemented by quantifying identified farm types in different districts. The intervention planning based on identified constraints for different components of farming systems including field crops and allied enterprises (livestock production, fish farming, bee keeping, etc.) in a systems perspective with multilevel interventions on the farmers’ fields could enable farmer to get multifold increase in net income.

## Supplementary Information

Supplementary Information.
