# Supplementary material for: Farm typology for planning targeted farming systems interventions for smallholders in Indo-Gangetic Plains of India
Source: Sci Rep. 2021 Oct 25;11:20978. doi: 10.1038/s41598-021-00372-w (PMC8546020; doi:10.1038/s41598-021-00372-w)
Supplement: Supplementary file 1 — Supplementary Information. [file 41598_2021_372_MOESM1_ESM.docx]

**Farm typology for planning targeted farming systems interventions for smallholders in Indo-Gangetic Plains of India**

Jashanjot Kaur^1^, A K Prusty^2*^, N Ravisankar^2^, A S Panwar^2^, M. Shamim^2^, S S Walia^1^, S Chatterjee^3^, M L Pasha^4^, Subhash Babu^5^, M L Jat^6^, Santiago López-Ridaura^7^, Jeroen C.J. Groot^8^, Roos Adelhart Toorop^8^, Luis Barba-Escoto^7^, Kohima Noopur^9^, Poonam Kashyap^2^

*Corresponding author: [aasiana143@gmail.com](mailto:aasiana143@gmail.com) (A. K. Prusty)

**Supplementary Information**

**Annexure- I**

|  | **Amritsar** | **Nadia** | **Kanpur** | **Meerut** | **Patiala** | **Purnia** | **Sirsa** | **Grand Total** |
| --- | --- | --- | --- | --- | --- | --- | --- | --- |
| Family size | 4 | 4 | 5 | 5 | 4 | 5 | 5 | 5 |
| Household head age | 42 | 52 | 47 | 53 | 46 | 41 | 40 | 46 |
| Family labour | 1.26 | 1.28 | 1.64 | 1.36 | 1.53 | 1.24 | 2.44 | 1.53 |
| Land owned | 1.20 | 0.74 | 0.87 | 0.99 | 1.39 | 0.78 | 0.70 | 0.96 |
| Land on rental basis | 0.07 | 0.12 | 0.00 | 0.15 | 0.00 | 0.00 | 0.00 | 0.05 |
| Land holding | 1.27 | 0.86 | 0.87 | 1.13 | 1.39 | 0.78 | 0.75 | 1.01 |
| Area with cereals | 163.74 | 97.41 | 165.56 | 49.06 | 165.10 | 169.30 | 99.22 | 130.16 |
| Area under fodders | 26.40 | 0.00 | 3.24 | 15.10 | 0.00 | 0.00 | 10.70 | 8.27 |
| Area under cash crops | 4.57 | 54.51 | 21.50 | 82.42 | 0.00 | 0.00 | 88.34 | 35.66 |
| Area under other crops | 0.00 | 27.67 | 2.98 | 2.99 | 0.00 | 15.49 | 0.00 | 6.56 |
| Total livestock units | 0.24 | 0.17 | 0.20 | 0.11 | 0.17 | 0.67 | 0.00 | 0.22 |
| Total number of local cattle | 2 | 1 | 1 | 1 | 2 | 2 | 1 | 1 |
| Total number of improved bred cattle | 1 | 1 | 0 | 0 | 1 | 0 | 0 | 1 |
| Total livestock | 3 | 2 | 2 | 2 | 2 | 2 | 2 | 2 |
| Total number of small ruminants | 0 | 0 | 1 | 0 | 0 | 3 | 0 | 1 |
| Total number of small animals | 0 | 1 | 0 | 0 | 0 | 1 | 0 | 0 |
| Milk production per animal | 1128 | 635 | 559 | 1186 | 1121 | 666 | 1355 | 952 |
| Total milk production | 4001 | 911 | 984 | 1910 | 2789 | 1427 | 2385 | 2074 |
| Income from crops | 74.36 | 83.28 | 67.86 | 68.25 | 79.97 | 63.04 | 60.17 | 70.74 |
| Income from livestock | 25.64 | 16.72 | 20.42 | 26.19 | 20.03 | 31.17 | 35.76 | 25.21 |
| Income from other sources | 0.0 | 0.0 | 11.7 | 5.6 | 0.0 | 5.8 | 4.1 | 4.0 |

**Annexure- II**

**NARP zone of and its agro-ecological features of studied districts in IGP**

| **District** | **NARP zone** | **Sub Region** | **Rainfall (in mm)** | **Climate** | **Soil** | **Major crops** |
| --- | --- | --- | --- | --- | --- | --- |
| Amritsar | PB-3 Central Plain Zone | Foothills of Shivalik & Himalayas | 890 | semi-arid to Dry sub-humid | Calcareous | Wheat, Rice, maize, sugarcane |
| Patiala | PB-3 Central Plain Zone | Plains | 561 | Semi-arid to Dry sub-humid | Alluvial (Recent) | Wheat, rice, maize, sugarcane |
| Nadia | New Alluvial Zone (WB-4) | Coastal Alluvial | 1385 | Dry humid to moist sub humid | Deep loamy soils | Rice, jute, sunflower |
| Sirsa | HR-2 Western Zone | Scarce Rainfall arid region | 360 | Arid and Extreme arid | Calcareous, Sierozemic, Alluvial (Recent), desert | Wheat, cotton, gram, Bajra, rice |
| Purnia | BI-2 North-east Alluvial Plain Zone | North-East Alluvial | 1470 | Dry sub-humid to moist sub-humid | Alluvial, tarai | Rice, wheat, maize, jute, gram |
| Kanpur | UP-4 Central Plain Zone | Central Plains | 979 | Dry sub-humid to semi arid | Alluvial | Wheat, rice, Arhar |
| Meerut | UP-1 Western Plain Zone | South-Western Plains | 721 | Semi-arid | Alluvial | Wheat, Bajra, rice,maize, Arhar, potato |

Source: dacnet.nic.in/farmer/new/dac/IndiaMap.asp (Accessed on 29 January 2019)
